# A Signaling Crosstalk Links SNAIL to the 37/67 kDa Laminin-1 Receptor Ribosomal Protein SA and Regulates the Acquisition of a Cancer Stem Cell Molecular Signature in U87 Glioblastoma Neurospheres

**DOI:** 10.3390/cancers14235944

**Published:** 2022-11-30

**Authors:** Loraine Gresseau, Marie-Eve Roy, Stéphanie Duhamel, Borhane Annabi

**Affiliations:** 1 Laboratoire d’Oncologie Moléculaire, Département de Chimie, and CERMO-FC, Université du Québec à Montréal, Montreal, QC H3C 3J7, Canada; 2 Goodman Cancer Institute, McGill University, Montreal, QC H3A 0G4, Canada

**Keywords:** glioblastoma, EGCG, cancer stem cells, spheroids, SNAIL, RPSA, laminin receptor, EMT

## Abstract

**Simple Summary:**

In vivo studies have shown that 3D neurosphere formation is a significant predictor of clinical outcome in glioma patients, and is a robust, independent predictor of glioma tumor progression. However, neurosphere assays, in common with other in vitro assays, are associated with some limitations. Little is known about the upstream signaling events triggered and that lead to a cancer stem cell molecular signature, even less is known on how anticancer diet-derived molecules can target such events. Here, we provide assessment of several signal transducing events involved in the acquisition of an in vitro stemness phenotype, in comparison to 2D monolayer cultures of brain cancer cells. We further identify a new signaling axis that can become a target for future therapies.

**Abstract:**

**Background**: Three-dimensional in vitro neurospheres cultures recapitulate stemness features associated with poor clinical outcome in glioblastoma patients. They are commonly used to address brain cancer stem cell (CSC) signal transducing biology that regulates spheroids formation and stemness phenotype, and to assess the in vitro pharmacological impact of chemotherapeutic drugs. **Objective**: Here, we addressed the role of a new signaling axis involved in the regulation of in vitro spheroids formation and assessed the chemopreventive ability of diet-derived epigallocatechin gallate (EGCG) to impact the processes that govern the acquisition of spheroids CSC stemness traits. **Methods**: Neurospheres were generated from adherent human U87 glioblastoma cancer cell cultures under conditions that recapitulate stemness features. Total RNA and protein lysates were isolated for gene expression by RT-qPCR and protein expression by immunoblot. Transcriptomic analysis was performed through RNA-Seq. **Results**: Compared to their parental adherent cells, tumorspheres expressed increased levels of the CSC markers *NANOG*, *SOX2*, *PROM1* (CD133), as well as of the epithelial-to-mesenchymal transition (EMT) markers *Fibronectin*, *SNAI1*, and 37/67 kDa laminin-1 receptor ribosomal protein SA (*RPSA*). Increased *PROM1*, *SOX2*, *Fibronectin*, and *RPSA* transcripts level were also observed in clinical grade IV glioblastoma tissues compared to normal tissue. EGCG treatment reduced dose-dependently tumorspheres size and inhibited the transcriptional regulation of those genes. An apoptotic signature was also found in spheroids with increased signal transducing events involving GSK3α/β, RSK, and CREB. These were repressed upon *RPSA* gene silencing and partially by *SNAI1* silencing. **Conclusion**: This work highlights a signaling axis linking RPSA upstream of SNAIL in neurospheres genesis and supports the chemopreventive impact that diet-derived EGCG may exert on the acquisition of CSC traits.

## 1. Introduction

The most dangerous primary brain tumour in adults with a WHO grade 4 is glioblastoma (GBM), which increases mortality in individuals with a 15-month median survival time [1,2]. The strong vascularization capability of GBM is consequent to adaptative molecular mechanisms, which supplies nutrients and oxygen to tumour core cells exposed to hypoxia and nutrient deprivation, promotes rapid tumour growth and spread. The blood–brain barrier remains one of the main barriers to getting anti-angiogenic chemotherapy medications into the brain tissue [3]. In addition, resistance to standard of care therapies including chemotherapy and radiotherapy, is thought to be responsible for brain cancer recurrence and metastasis [4]. This resistance is also in part attributable to cancer-initiating/cancer stem-like cells, defined as a small highly malignant subpopulation of cancer cells that are endowed with higher tumor-initiating ability.

Strategies to prevent the acquisition of cancer stemness or to target brain cancer stem cells (CSC) to overcome therapy resistance have recently led to innovative diet-derived approaches to prevent tumor relapse [5]. Epigenetic diet approaches against CSC are, among the recent research avenues, emerging as a very new strategy with a good future expectation to treat cancer patients [6]. The study of epigenetics is also particularly interested in several bioactive food ingredients. Numerous substances exhibit anticancer effects through epigenetic targets and may aid in the prevention of cancer. Dietary substances may alter normal epigenetic states and reverse aberrant gene activation or silencing [7]. Natural substances, mostly phytochemicals, have recently drawn a lot of attention due to their wide range of safety profiles, capacity to target both CSC and diverse populations of cancer cells, and critical signalling pathways [8].

Small numbers of cells that are the source of primary malignant tumours undergo mutations in oncogenes, tumour suppressors, or DNA repair genes to become extremely proliferative [9]. Even while genetic abnormalities eventually result in the development of massive, intricate vascular tumours, they all initially undergo an avascular (blood vessel-free) hypoxic mode of growth [10]. Understanding this stage of growth is important for comprehending how tumours behave at later stages as well as the contribution of CSC. Spheroids of tumours are frequently employed as in vitro models of avascular tumour growth because they are thought to recapitulate the CSC phenotype [11]. They are spherical aggregations of cancer cells that have a controlled concentration of nutrients added to them. This makes it possible to quantitatively study the effects of spheroid size, cell counts, and proportions of hypoxic, quiescent, and proliferative cells on CSC phenotypic acquisition [12].

Epigallocatechin-3-gallate (EGCG), a biological active polyphenol found in green tea leaves, can suppress brain cancer cell proliferation, and induce apoptosis [13]. Its specific effects on stemness traits in brain malignancies remain however unclear. It is therefore mandatory to explore the chemopreventive properties of EGCG in targeting CSC proliferation and survival [14,15]. The role of polyphenols in overcoming cancer drug resistance has also been inferred [16]. EGCG has been shown as a potent inhibitor of U87 glioblastoma cell growth and these effects are in most cases mediated by apoptosis [17]. Whether these properties impact the acquisition of a CSC phenotype is unknown.

Here, we generated an in vitro grade-4 brain cancer spheroid model from a commercially available U87 glioblastoma cell line model. Transcriptomic analysis confirmed the increased expression of CSC-associated genes recapitulating in part CSC-like characteristics in brain cancer cells. Among those genes are CSC biomarkers, epithelial-to-mesenchymal transition (EMT) and integrin signaling, that contribute to maintain an undifferentiated and pluripotent state, while others are involved in cell motility, self-renewal and chemoresistance. We further highlighted a new role of a signaling axis involving a 37/67 kDa laminin receptor ribosomal protein SA (RPSA) upstream of SNAIL in neurospheres formation that regulates a combined apoptotic/EMT/CSC phenotype involved in chemoresistance and invasion and that can be targeted by EGCG.

## 2. Materials and Methods

### 2.1. Materials

Sigma-Aldrich Corp provided the bovine serum albumin (BSA) and sodium dodecyl sulphate (SDS) (St. Louis, MO, USA). We purchased cell culture medium from Life Technologies Corp (Carlsbad, CA). Chemicals for electrophoresis were bought from Bio-Rad Laboratories (Hercules, CA, USA). Denville Scientific Inc. (Holliston, MA, USA) provided the HyGLO^TM^ Chemiluminescent HRP (horseradish peroxidase) Antibody Detection Reagents (Metuchen, NJ, USA). Thermo Fisher Scientific (Waltham, MA, USA) sells the Micro BCA^TM^ Protein Assay Kit. The polyclonal antibodies against PARP (#9542), BCL-2 (50E3, #2870), BCL-XL (54H6, #2764P), and PI3K were all from Cell Signaling Technology, as were the monoclonal antibodies against pAKT (Ser473) (D9W9U, #12694). The monoclonal antibody against the 90 kDa ribosomal S6 kinases (RSK1/RSK2/RSK3) (32D7, #9355), phosphorylated p90RSK (ser380), and the polyclonal antibodies against Glycogen Synthase Kinase-3 (GSK3)-α/β (D75D3, #5676), phosphorylated (P)-GSK3-α/β Danvers, MA, USA). R&D Systems Inc. (Minneapolis, MN, USA) was used to acquire monoclonal antibodies against human Mitogen- and stress-activated protein kinase 1 (MSK1, S376, #AF2518), phosphorylated (P)-MSK1 (#MAB1094), and MSK2 (S360, #MAB2310) (Minneapolis, MN, USA). Jackson ImmunoResearch Laboratories provided the donkey anti-rabbit and anti-mouse immunoglobulin (Ig) G secondary antibodies (West Grove, PA, USA). EGCG was from MP Biomedicals (Santa Ana, CA, USA). The rest of the chemicals were from Sigma-Aldrich Corp.

### 2.2. Cell Culture

American Type Culture Collection provided human U87 glioblastoma cells (ATCC; Manassas, VA, USA). Serum starvation was achieved using Eagle’s minimal essential medium (EMEM; Gibco BRL, Grand Island, NY, USA), 10% inactivated foetal bovine serum (Hyclone Laboratories, Logan, UT, USA), and 100 units/mL penicillin/streptomycin. Cells were cultured at 37 °C under a humidified 95%/5% (*v*/*v*) mixture of air and CO_2_. U87 neurosphere formation was performed as follows: 80–90% adherent U87 monolayer cells were trypsinized and plated in low adhesion 24-well plates (Corning Costar, Corning, NY, USA) at a density of 2 × 10^5^ cells/mL in complete media for 24–72 h. Then, the supernatant was removed, and serum-free EMEM supplemented with 10 ng/mL human basic fibroblast growth factor (Gibco, Thermo Fisher, 13256029), 20 ng/mL human epidermal growth factor (Gibco, Thermo Fisher, PHG0315), 5 μg/mL insulin (Sigma Aldrich Corp, I3536,) and bovine serum albumin (BSA) (Sigma Aldrich Corp, A9418-5G,) at 4% was carefully added to the dishes. Spheroids were defined as rounded aggregates of cells with a smooth surface and poor cell-to-cell definition. Perimeter of 30–70 spheroids/flask were assessed for each experimental condition performed in triplicate and derived from three independent experiments. For 4× magnification, the following calculation was performed: One (1) pixel = 6.4 µm/(4× * 0.67) = 2.39 µm (0.00239 mm/pixel). Thus, number of pixels in 1 mm = 1 mm/0.00239 mm/pixel = 418 pixels. For 10× magnification, replace 4× in the formula. Cells were cultured at 37 °C under a humidified 95%/5% (*v*/*v*) mixture of air and CO_2_.

### 2.3. Total RNA Isolation, cDNA Synthesis, and Real-Time Quantitative PCR

According to the manufacturer’s instructions, total RNA was extracted from cell monolayers or tumorspheres using 1 mL of TriZol reagent for up to 3 × 10^6^ cells (Life Technologies, Gaithersburg, MD, USA). A high-capacity cDNA reverse transcription kit (Applied Biosystems, Foster City, CA, USA) or, in the case of the gene array, the R2 First Strand kit was used to reverse-transcribe 1 μg of total RNA for cDNA synthesis (QIAGEN, Valencia, CA, USA). Before PCR, the cDNA was kept at −80 °C. iQ SYBR Green Supermix was used in real-time quantitative PCR to measure gene expression (Bio-Rad, Hercules, CA, USA). Utilizing a Bio-Rad Icycler iQ5 for DNA amplification, product identification was carried out by detecting the binding of fluorescent dye SYBR Green I to double-stranded DNA. The following primer sets were from QIAGEN: GAPDH (Hs_GAPDH_1_SG, QT00079247), Peptidylprolyl Isomerase A (PPIA) (Hs_PPIA_4_SG, QT01866137), β-Actin (Hs_Actb_2_SG, QT01680476), SNAI1 (Hs_SNAI1_1_SG, QT00010010), Fibronectin (Hs_FN1_1_SGQT00038024), CD133 (Hs_PROM1_1_SG, QT00075586), NANOG (Hs_NANOG_2_SG, QT01844808), SOX2 (Hs_SOX2_1_SG, QT00237601), and RPSA (Hs_RPSA_1_SG, QT00044310). The relative quantities of target gene mRNA were normalized against internal housekeeping genes *PPIA* and *GAPDH*. The RNA was measured by following a ∆C_T_ method employing an amplification plot (fluorescence signal vs. cycle number). The difference (∆C_T_) between the mean values in the triplicate samples of the target gene and the housekeeping genes was calculated with the CFX manager Software version 2.1 (Bio-Rad) and the relative quantified value (RQV) was expressed as 2^−∆CT^.

### 2.4. Total RNA Library Preparation

Total RNA (500 ng) was extracted from U87-treated cells and used for library preparation. RNA quality was assessed with the Bioanalyzer RNA 6000 Nano assay on the 2100 Bioanalyzer system (Agilent Technologies, Mississauga, ON, Canada), and all samples had a RNA integrity number (RIN) above eight. Library preparation was carried out with the KAPA mRNA-Seq HyperPrep kit (KAPA, Cat no. KK8581). Ligation was made with Illumina dual-index UMI, and 10 PCR cycles were required to amplify cDNA libraries. Libraries were quantified by QuBit and BioAnalyzer DNA1000. All libraries were diluted to 10 nM and normalized by qPCR using the KAPA library quantification kit (KAPA; Cat no. KK4973). Libraries were pooled to equimolar concentrations. Three biological replicates were generated.

### 2.5. RNA Sequencing

High RNA quality was verified as above, and samples were sequenced using the Illumina NextSeq500 sequencer at the Genomics Core Facility of the Institute of for Research in Immunology and Cancer (IRIC, Montreal, QC, Canada).

### 2.6. Reads Alignment and Differential Expression Analysis

Reads were aligned and sorted by coordinates to the human genome build 38 (GRCh38.p13) with version 37 of Gencode gene annotations, using the STAR aligner (STAR_2.7.1a) [18,19]. Quantification of genes was performed during alignment by STAR. Differentially expressed genes among groups were identified using the R packages DESeq2 (v 1.30.1) [20]. After analysis, only genes with adjusted *p*-values (adjp) < 0.05, and log2 fold change ≥1.0 were considered as significant. Hierarchical clustering of differentially expressed genes was used to represent the results (R package ggplot2).

### 2.7. Gene Set Enrichment Analysis

The gene set enrichment analysis was performed with the GSEA software version 4.2.3 [21] with the complete set of normalized input values, using the Hallmark, canonical pathway gene sets (chemical and genetic perturbations, BioCarta, Reactome, and Kegg), and Gene ontology gene sets (Biological process). The software R (version >3.4) was used for data analysis. For all statistical analyses, differences were considered statistically significant if the adjp calculated by Student’s *t* test with Bonferroni correction were <0.05. RNA-Sequencing of the U87 cell lines.

### 2.8. Western Blot

Cells were lysed in a buffer containing 1 mM each of NaF and Na_3_VO_4_, and proteins (10–20 µg) were separated by SDS-polyacrylamide gel electrophoresis (PAGE). Proteins were then electro-transferred to polyvinylidene difluoride membranes and blocked with 5% nonfat dry milk in Tris-buffered saline (150 mM NaCl, 20 mM Tris-HCl, pH 7.5) containing 0.3% Tween-20 for an hour at room temperature (TBST; Bioshop, TWN510-500). After being thoroughly cleaned in TBST, the membranes were incubated with the appropriate primary antibodies (1/1000 dilution) in TBST containing 3% BSA and 0.1% sodium azide (Sigma-Aldrich) overnight at 4 °C in a shaker. The membranes were treated with horseradish peroxidase-conjugated anti-rabbit or anti-mouse IgG at 1/2500 dilutions for 1 h in TBST containing 5% nonfat dry milk after three TBST washes. ECL was used to make immunoreactive material visible.

### 2.9. In Silico Analysis of Transcripts Levels in Clinical Glioblastoma and Low-Grade Glioma Tissues

A Gene Expression Profiling Interactive Analysis (GEPIA) web server was used to analyze the RNA sequencing expression data of GBM tumors (*n* = 163) vs. healthy tissue (*n* = 207), and of low-grade glioma (*n* = 251) vs. healthy tissue (*n* = 207) from the TCGA and the GTEx projects was used [22]. GEPIA provides customizable functions such as tumor/normal differential expression analysis, profiling according to cancer types or pathological stages, patient survival analysis, similar gene detection, correlation analysis and dimensionality reduction analysis (http://gepia.cancer-pku.cn/detail.php, accessed on 15 July 2022).

### 2.10. Statistical Data Analysis

Unless otherwise stated, data and error bars were expressed as the mean standard error of the mean (SEM) of three or more separate experiments. The Kruskal–Wallis test was used to assess hypotheses, followed by a Mann–Whitney test or a Dunn-Tukey post-test (for data with more than three groups) (two group comparisons). Probability values of 0.05 or 0.01 were judged significant and indicated in the figures as (*) or (**), respectively. The statistical analysis software GraphPad Prism 7 was used for all calculations (San Diego, CA, USA).

## 3. Results

**Figure 1:**
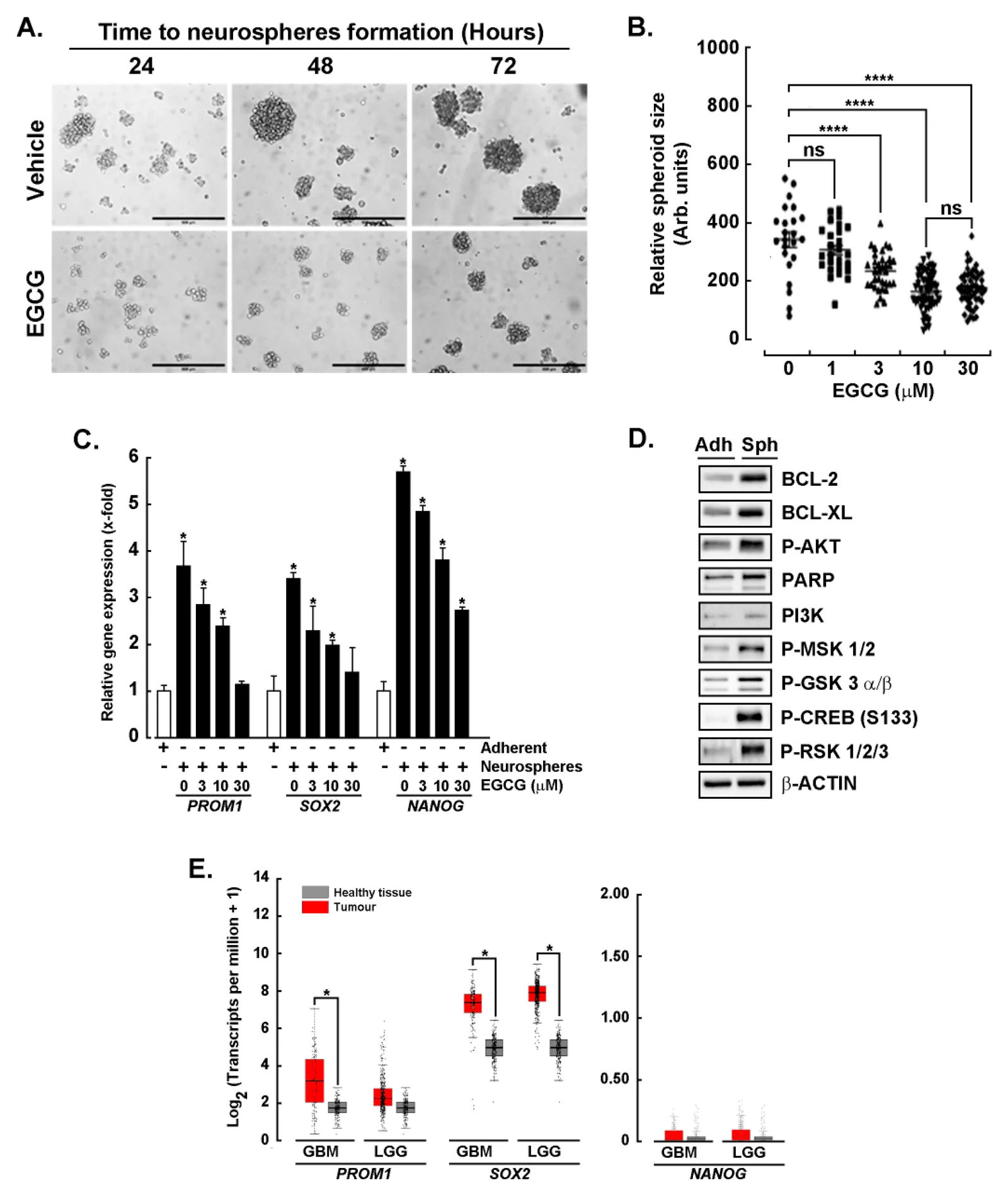
EGCG alters neurospheres size and reverses the acquisition of a cancer-stem cell phenotype. (**A**) U87 glioblastoma monolayers were cultured with the Tumorsphere Medium Xf with SupplementMix for the indicated times in the absence or presence of increasing EGCG concentrations and spheroids. Representative phase contrast pictures were taken, and (**B**) spheroid size was quantified as described in the Methods section (**** = 0.0001 ≥ *p;* ns = non significant) (**C**) Total RNA was extracted from adherent or neurospheres generated upon 72 h in the presence of increasing EGCG concentrations. RT-qPCR analysis was used to assess the expression of CSC markers *PROM1*, *SOX2*, and *NANOG*. Probability values of 0.05 were judged significant and indicated as (*). (**D**) Cell lysates were harvested from adherent, or spheroids obtained at 72 h. Western blot analysis of protein expression was performed as described in the Methods section of the described markers. Representative blots for each marker are shown from three independent experiments. (**E**) In silico analysis of *PROM1*, *SOX2*, and *NANOG* transcript levels was performed on clinical samples from GBM, and low-grade glioma (LGG) and compared to healthy tissue as described in the Methods section. Probability values of 0.05 were judged significant and indicated as (*).

EGCG alters neurospheres size and reverses the acquisition of a cancer-stem cell molecular signature. Generation of U87 glioblastoma neurospheres culture conditions were first validated. Neurospheres appeared to reach maturation at 72 h (Figure 1A, upper panels), whereas the addition of EGCG significantly impede their growth (Figure 1A, lower panels) with minimal 1.4–2.9% cytotoxic impact (Supplementary Section, Figure S1). The inhibitory effect of EGCG on spheroids size was found dose-dependent and maximally inhibited between 10–30 μM (Figure 1B). In order to monitor molecular signature changes regarding the acquisition of stemness traits, total RNA was extracted from adherent U87 cells or neurospheres generated upon 72 h in the presence of increasing EGCG concentrations. First, RT-qPCR analysis revealed that the expression of the CSC markers *PROM1*, *SOX2*, and *NANOG* was increased in neurospheres compared to adherent U87 cells. Notably, EGCG was sufficient to reduce the expression of these markers dose-dependently (Figure 1C). Next, cell death resistance and pro-survival signaling pathways were investigated at the protein level in cell lysates upon spheroids formation. The expression of the anti-apoptotic proteins Bcl-2 and Bcl-xL was induced in neurospheres in accordance with previous reports [23,24]. Interestingly, this was also accompanied by increased expression of the pro-survival phosphorylated Akt (Figure 1D). All uncropped Western blots can be found in the Supplementary Section (Figure S2). Additionally, in silico differential analysis of *PROM1*, *SOX2*, and *NANOG* transcript levels was performed on clinical samples from GBM and low-grade glioma (LGG) and compared to healthy tissues as described in the Methods section. Except for *NANOG*, which expression was not significantly changed, both *PROM1* and *SOX2* expression were found significantly higher in GBM samples compared to healthy tissue (Figure 1E, red boxes), while only *SOX2* was increased in LGG (Figure 1E, grey boxes). This appears suggestive of a cascade of adaptative events that may occur during the acquisition of a more aggressive phenotype during brain cancer. Altogether, the acquisition/expression of stem cell molecular signature is recapitulated upon spheroid formation and suggests that such phenotype be more present in higher grades of GBM.

**Figure 2:**
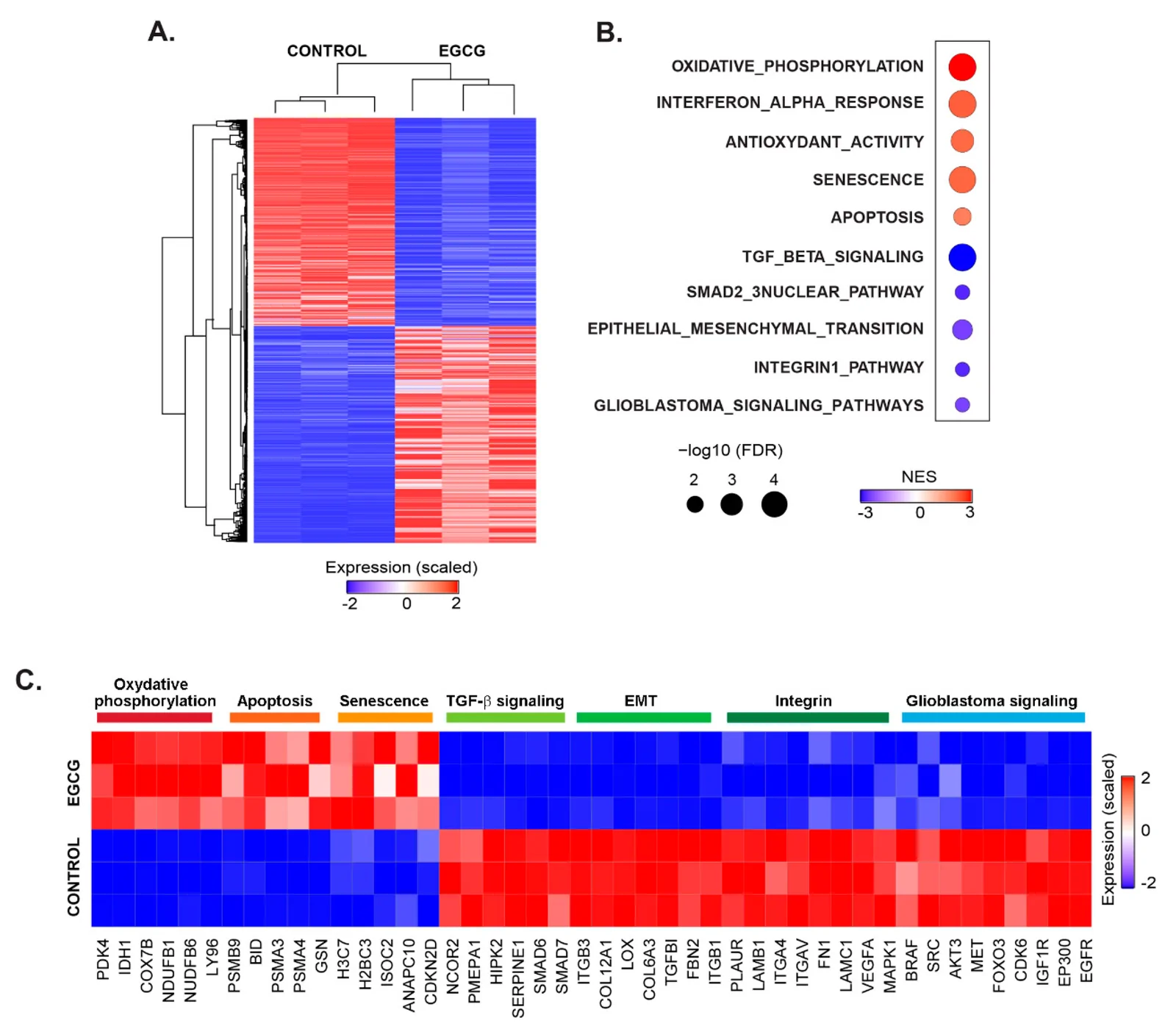
Transcriptomic analysis reveals the reversion of epithelial to mesenchymal transition in profit to an apoptotic program upon EGCG treatment of glioblastoma spheroids. (**A**) Total RNA was extracted from control- and EGCG- treated U87 spheroids from three independent experiments and subjected to RNA-Sequencing. Unsupervised hierarchical clustering of all the genes differentially expressed in pairwise comparison was tested with adjusted *p* value ≤ 0.05 and log2 fold change ≥1.0. (**B**) Dot plot showing changes in the normalized enrichment score (NES) for the most enriched pathways in EGCG-treated U87 spheroids compared to untreated control cells. FDR *p* value ≤ 0.05. The top up- and down-regulated gene signatures of Hallmark, Canonical pathways, and Gene Ontology. (**C**) Heatmap representation of the relative expression of known phenotypic markers for each indicated cellular process.

Transcriptomic analysis reveals the reversion of epithelial-to-mesenchymal transition for the benefit of an apoptotic, senescent, and oxidative program in EGCG-treated neurospheres. To gain further insight into the differential transcription regulation that EGCG exerts against U87 glioblastoma neurospheres genesis, total RNA was isolated from untreated or EGCG-treated neurospheres, and RNA-Sequencing was performed as described in the Methods section. Unsupervised hierarchical clustering of significantly altered transcripts (genes with adjusted *p*-value ≤0.05 and log2 fold change ≥ 1.0) revealed that neurospheres generated upon EGCG treatment displayed a gene expression signature distinct from that of control neurospheres. Globally, EGCG treatment led to the significant increase of 1285 genes and the reduction of 1196 genes (Figure 2A). To gain further understanding of these differences, we performed gene set enrichment analysis (GSEA). Among the processes found to be significantly induced in EGCG-treated neurospheres, these included oxidative phosphorylation, senescence, and apoptosis (Figure 2B, red dots). This was associated with a significant increase in the transcript levels of the pro-apoptotic regulator BID and several proteasome catalytic subunit family members (PSMA3, PSMA4, PSMB3, and PSMB9). The Cyclin-Dependent Kinase Inhibitor 2D (CDKN2D) which encodes for p19INK4D, an essential regulator of apoptosis [25], DNA damage repair, and senescence were also significantly enhanced. Notably, EGCG increased the transcriptional level of gelsolin (GSN), previously reported to impede the malignant phenotype of GBM by reducing cell proliferation and invasive properties (Figure 2C) [26].

Negative enrichment in TGF-β, SMAD2/3, and epithelial-to-mesenchymal transition (EMT) gene signatures were observed upon EGCG treatment (Figure 2B, blue dot). This corroborated the repression of the SMAD family members (*SMAD3*, *SMAD6*, and *SMAD7*) and *TGFBI* transcripts. Accordingly, the expression of several markers of EMT, such as Fibronectin (*FN*), collagens (*COL12A1* and *COL6A3*), and lysyl oxidase (*Lox*), was significantly reduced in the EGCG-treated neurospheres (Figure 2C). Importantly, we report for the first time to our knowledge that EGCG led to the transcriptional downregulation of integrin type 1 family members *ITGB1*, *ITGA4*, and *ITGAV*. These integrins have been related with a mesenchymal subset of GBM associated with poor clinical outcome [27] and bevacizumab resistance [28]. Negative enrichment for GBM signaling pathways was also observed upon EGCG treatment. The transcriptional levels of the RTKs *MET* and *EGFR*, as well as *BRAF* and *MAPK1* was reduced. The expression of cyclin dependent-kinases 6 (*CDK6*), *SRC*, and *AKT3* was also highly decreased in response to EGCG. Several studies reported that the CDK4/6 axis and RTK signaling are concomitantly deregulated in GBM, creating an opportunity to develop more effective therapies by targeting both pathways [29,30,31]. Moreover, activation of the Met/TrkA-B pathway is involved in therapeutic resistance of GBM to CDK4/6 inhibitors, suggesting that dual inhibition of c-Met/Trk and CDK4/6 should be considered in resistant disease [30]. In conclusion, EGCG reduces the TGF-β/SMAD2/3 signaling pathways impeding EMT and appears to support the induction of an apoptotic program in GBM cells.

**Figure 3:**
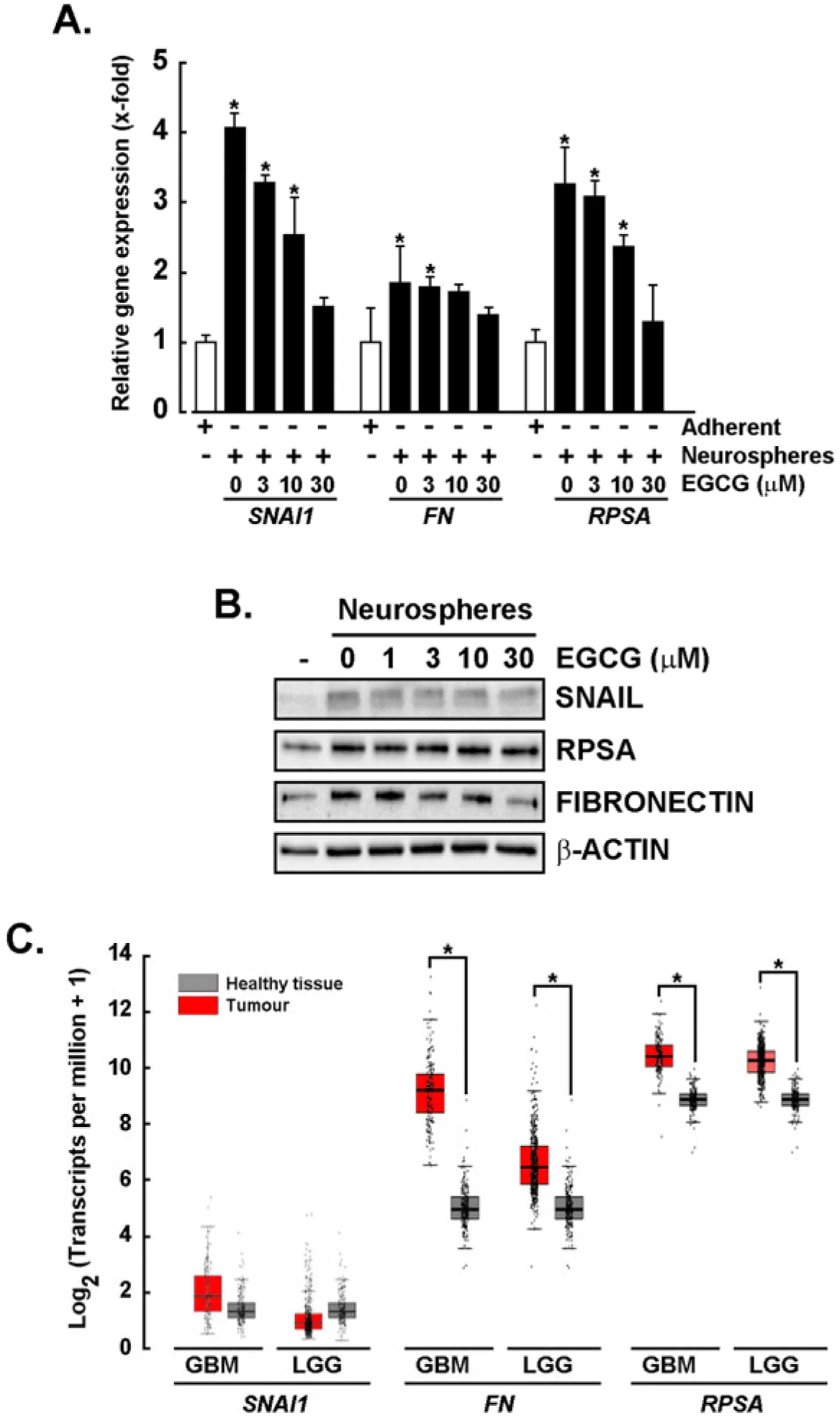
EGCG alters neurospheres’ epithelial-to-mesenchymal transition phenotype. (**A**) U87 glioblastoma monolayers were cultured with the Tumorsphere Medium Xf with SupplementMix to generate spheroids for 72 h in the absence or presence of increasing EGCG concentrations. Total RNA was extracted from adherent or neurospheres, and RT-qPCR analysis was used to assess the expression of EMT markers SNAIL, Fibronectin (FN), and RPSA (67 kDa Laminin Receptor). (**B**) Cell lysates were harvested from adherent (Adh), or neurospheres obtained at 72 h. Western blot analysis of protein expression was performed as described in the Methods section of the described markers. Representative blots for each marker are shown from three independent experiments. (**C**) In silico analysis of *SNAI1*, *FN*, and *RPSA* transcript levels was performed on clinical samples from GBM, and low-grade glioma (LGG) and compared to healthy tissue as described in the Methods section. Probability values of 0.05 were judged significant and indicated in the figure as (*).

EGCG alters neurospheres’ epithelial-to-mesenchymal transition phenotype. Our unbiased transcriptomic analysis revealed a substantial decrease in EMT and stem-like features upon EGCG treatment of neurospheres. Further investigations pointed to several regulators of cell adhesion and extracellular matrix interaction, including integrins and laminins whose expression was also decreased (Figure 2B,C). *LAMC1* was among the most significantly downregulated gene in the EGCG-treated spheroids. The impact of EGCG was next assessed on the expression of EMT markers SNAIL, FN, and 37/67 kDa Laminin Receptor Protein SA (RPSA). U87 glioblastoma neurospheres were generated in the absence or presence of increasing EGCG concentrations. Total RNA was extracted from adherent or neurospheres, and EMT markers expression was assessed at both the gene and protein levels by RT-qPCR (Figure 3A) and Western blotting (Figure 3B), respectively. A similar impact of EGCG was observed at both the transcriptional and translational levels as SNAIL, FN, and RPSA were collectively induced upon neurospheres formation, while their expression was dose-dependently decreased by increasing EGCG concentrations. All uncropped Western blots can be found in the Supplementary Section (Figure S3). Again, in silico analysis of *SNAI1*, *FN*, and *RPSA* transcript levels was performed on clinical samples from GBM and LGG. Except for *SNAI1*, which expression levels were not higher in either GBM or LGG when compared to healthy tissue, both FN and RPSA expression were found significantly higher in GBM and in LGG samples (Figure 3C). Such profiling of EMT biomarkers mimics that observed upon spheroids formation.

**Figure 4:**
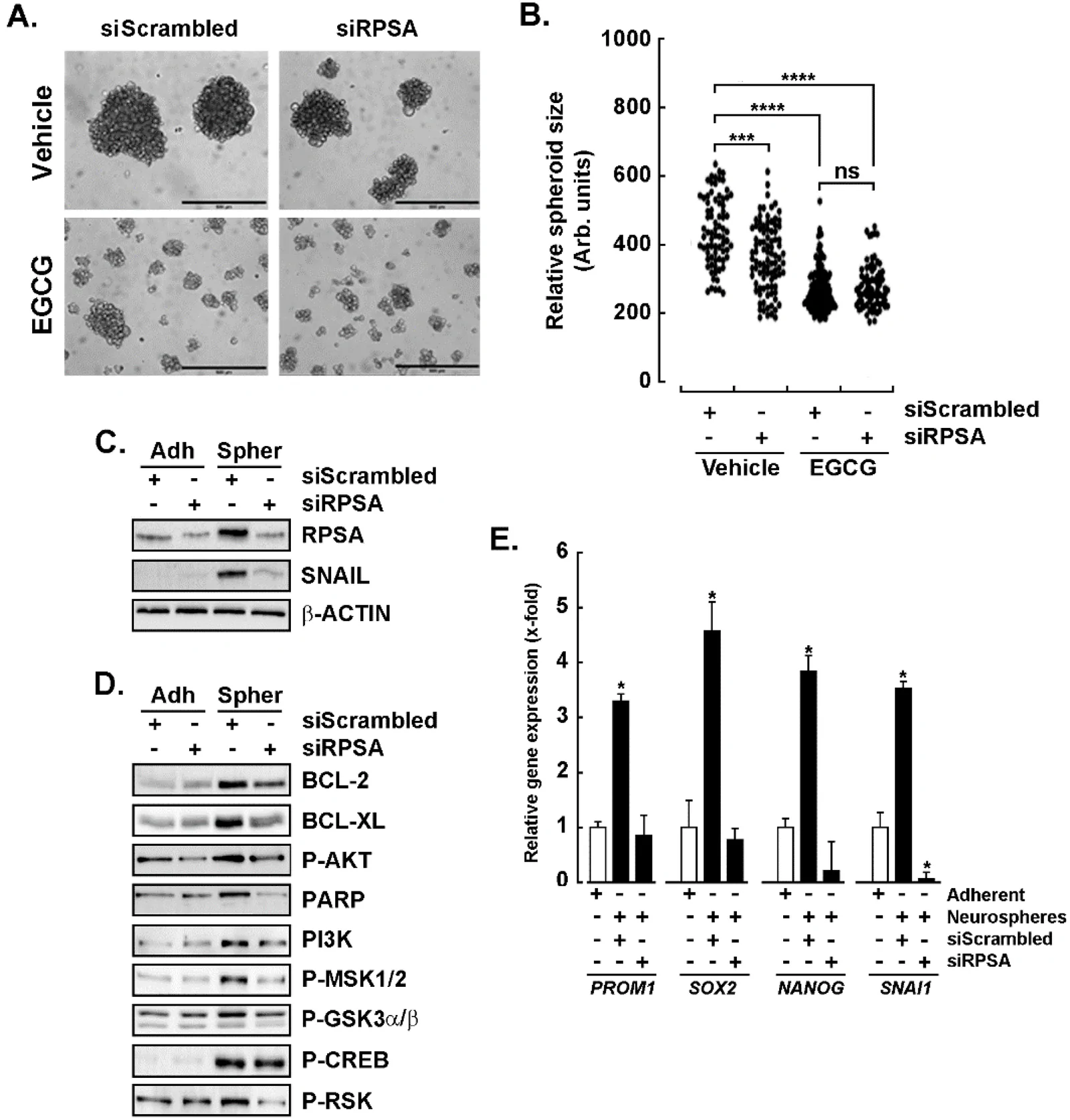
Repression of RPSA alters spheroids formation and prevents the acquisition of a cancer stem cell phenotype. (**A**) U87 glioblastoma monolayers were transiently transfected with a scrambled sequence (siScrambled) or with a siRNA directed against RPSA (siRPSA). Next, the cells were cultured with the Tumorsphere Medium Xf with Supplement Mix for 72 h in the absence or presence of 30 μM EGCG. Representative phase contrast pictures of the spheroids formed were taken, and (**B**) spheroid size was quantified as described in the Methods section. (*** = 0.001 ≥ *p*; **** = 0.0001 ≥ *p*; ns = non significant). Cell lysates were harvested from adherent, or spheroids obtained at 72 h and upon transient siScrambled or siRPSA transfection. Western blot analysis of (**C**) EMT markers RPSA, SNAIL, and β-Actin, as well as the indicated (**D**) apoptosis and transducing intermediates expression was performed as described in the Methods section. Representative blots for each marker are shown from three independent experiments. (**E**) Total RNA was extracted from adherent or neurospheres and RT-qPCR analysis was used to assess the expression of CSC markers (*PROM1*, *SOX2*, and *NANOG*), and EMT marker *SNAI1*. Probability values of 0.05 were judged significant and indicated as (*).

Repression of the 37/67 kDa laminin-1 receptor ribosomal protein SA (RPSA) alters spheroids formation and prevents the acquisition of a cancer stem cell phenotype. Given the significantly increased RPSA transcript and protein levels in neurospheres as well as in GBM and LGG clinical samples (Figure 3), the functional impact of RPSA was next assessed. U87 glioblastoma monolayers were transiently transfected with a scrambled sequence (siScrambled) or with a siRNA directed against RPSA (siRPSA), then neurospheres generated in the absence or presence of 30 μM EGCG (Figure 4A). Spheroid size was found decreased to relatively the same extent between siRPSA and EGCG-treated cells (Figure 4B). Cell lysates were harvested from adherent, or spheroids obtained upon transient siScrambled or siRPSA transfection. Western blot analysis of EMT markers and RPSA confirmed the silencing efficiency, but also that SNAIL expression induction was decreased consequent to reduced RPSA expression (Figure 4C), as well as that of Fibronectin (Supplemental data, Figure S4). Induced apoptosis and transducing phosphorylated intermediates expression was also found altered, except for CREB (Figure 4D). All uncropped Western blots can be found in the Supplementary Section (Figure S5). Total RNA was extracted from adherent cells and neurospheres, and RT-qPCR analysis revealed that the expression of the CSC markers *PROM1*, *SOX2*, and *NANOG*, and EMT marker *SNAI1* was repressed (Figure 4E).

**Figure 5:**
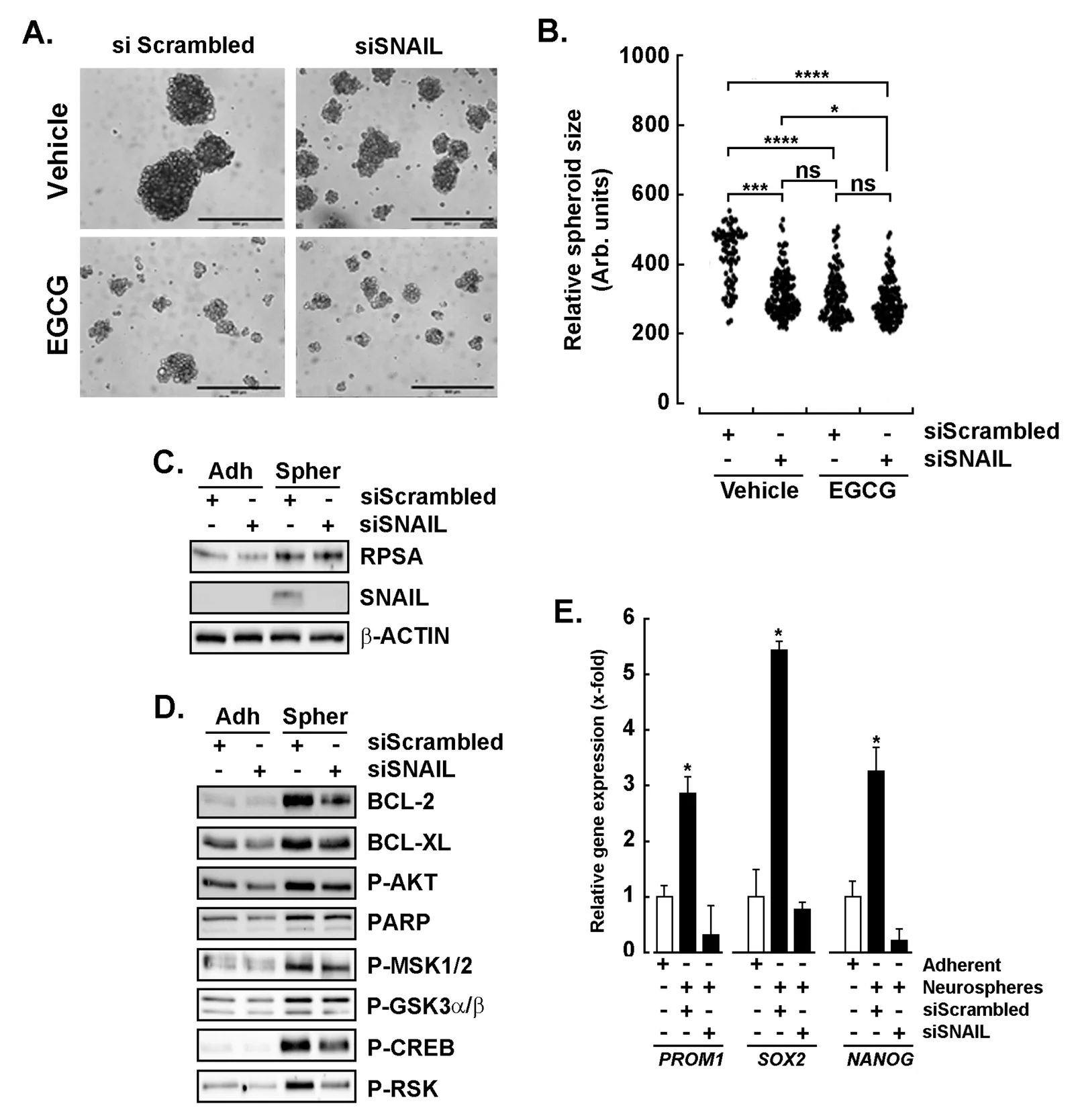
Repression of SNAIL alters spheroids formation and prevents the acquisition of a cancer stem cell phenotype but does not affect the acquisition of the apoptotic signature. (**A**) U87 glioblastoma monolayers were transiently transfected with a scrambled sequence (siScrambled) or with a siRNA directed against SNAIL (siSNAIL). Next, the cells were cultured with the Tumorsphere Medium Xf with SupplementMix for 72 h in the absence or presence of 30 μM EGCG. Representative phase contrast pictures of the spheroids formed were taken, and (**B**) spheroid size was quantified as described in the Methods section. (*** = 0.001 ≥ *p*; **** = 0.0001 ≥ *p*; ns = non significant). Cell lysates were harvested from adherent, or spheroids obtained at 72 h and upon transient siScrambled or siSNAIL transfection. Western blot analysis of (**C**) EMT markers RPSA, SNAIL, and β-Actin, as well as the indicated (**D**) apoptosis and transducing intermediates expression was performed as described in the Methods section. Representative blots for each marker are shown from three independent experiments. (**E**) Total RNA was extracted from adherent or neurospheres, and RT-qPCR analysis was used to assess the expression of CSC markers (PROM1, SOX2, and NANOG). Probability values of 0.05 were judged significant and indicated as (*).

Repression of SNAIL alters spheroids formation, prevents the acquisition of a cancer stem cell phenotype, but does not affect the acquisition of the apoptotic signature. Given the apparent signaling axis linking RPSA to SNAIL, the functional impact of SNAIL was next assessed. U87 glioblastoma monolayers were transiently transfected with a scrambled sequence (siScrambled) or with a siRNA directed against SNAIL (siSNAIL), then neurospheres generated in the absence or presence of 30 μM EGCG (Figure 5A). Spheroid size was quantified and found decreased to relatively the same extent between siSNAIL and EGCG-treated cells (Figure 5B). Cell lysates were harvested from adherent, or spheroids obtained upon transient siScrambled or siSNAIL transfection. Western blot analysis of SNAIL confirmed the silencing efficiency, but the induced RPSA expression found unaltered suggesting RPSA acts upstream of SNAIL (Figure 5C). In coherence, induced apoptosis and transducing phosphorylated intermediates expression was globally found unaltered, whereas only RSK phosphorylation was reduced (Figure 5D). All uncropped Western blots can be found in the Supplementary Section (Figure S6). Total RNA was extracted from adherent or neurospheres, and RT-qPCR analysis revealed that the gene expression of CSC markers *PROM1*, *SOX2*, and *NANOG*, was repressed similarly to siRPSA conditions (Figure 5E).

## 4. Discussion

It is hypothesised that lifestyle variables like smoking, drinking alcohol, and eating a high-calorie diet expand stem cell pools and induce CSC characteristics in malignancies [32]. The development of tumours and the spread of cancer may be aided by factors that increase the number of normal stem cells or encourage tumour cells to acquire stemness characteristics. Thus, understanding the impact of diet-derived molecules on the early signaling events that would prevent oncogenic transformation of normal stem cells could halt the emergence of CSC, although these can also originate from de-differentiation of bulk tumor cells. Diet-derived phenolic compounds could represent such a vast group of chemopreventive substances against the emergence of CSC with anticarcinogenic functions, anti-inflammatory, and antioxidative activities [33].

Glioblastomas frequently relapse following radiation or chemotherapy, suggesting that a fraction of therapy-resistant brain cancer cells remains responsible for tumor regrowth [34]. The recent identification of a brain CSC subpopulation with potent tumorigenic activity supports such therapy resistance phenotype, which molecular signature can be partially recapitulated in vitro in CD133-enriched neurospheres derived from glioblastoma primary cultures [35]. Interestingly, multivariate analysis of cell spheroids, tumor grade, and patient age demonstrated that neurosphere formation was a robust, independent predictor of glioma tumor progression [36]. While such CSC model suites the pharmacological studies on the impact of chemotherapeutic drugs on the phenotypic and molecular signature of spheroid cultures, less is understood when addressing the early upstream signal transducing events that dictate neurosphere generation and their targeting.

Here, we demonstrate that U87 glioblastoma 3D neurospheres culture conditions can partially recapitulate the CSC chemoresistance and invasive molecular signature by increasing *PROM1*, *SOX2*, and *NANOG* expression. In fact, CD133-positive glioblastoma cells were associated with an invasive phenotype [37], and high CD133 expression is associated with in vitro resistance to chemotherapy involving activation of the AKT pathway in neuroblastoma cell model [38]. In addition, we also observed increases in EMT markers such as SNAIL, FN, and RPSA, as well as the adaptative cell survival molecular signatures, including increases in anti-apoptotic Bcl-2 and Bcl-xL, and of the prosurvival AKT phosphorylation status. This is coherent when one considers that the formation of such avascular organoids is accompanied with an increased hypoxic microenvironment [39]. More importantly, exploiting EGCG’s chemopreventive properties in a coordinated intervention on these early events, which signal and lead to such CSC phenotype in neurospheres, appears to efficiently inhibit the adaptative hypoxia-to-EMT processes that lead to a CSC phenotype.

**Figure 6:**
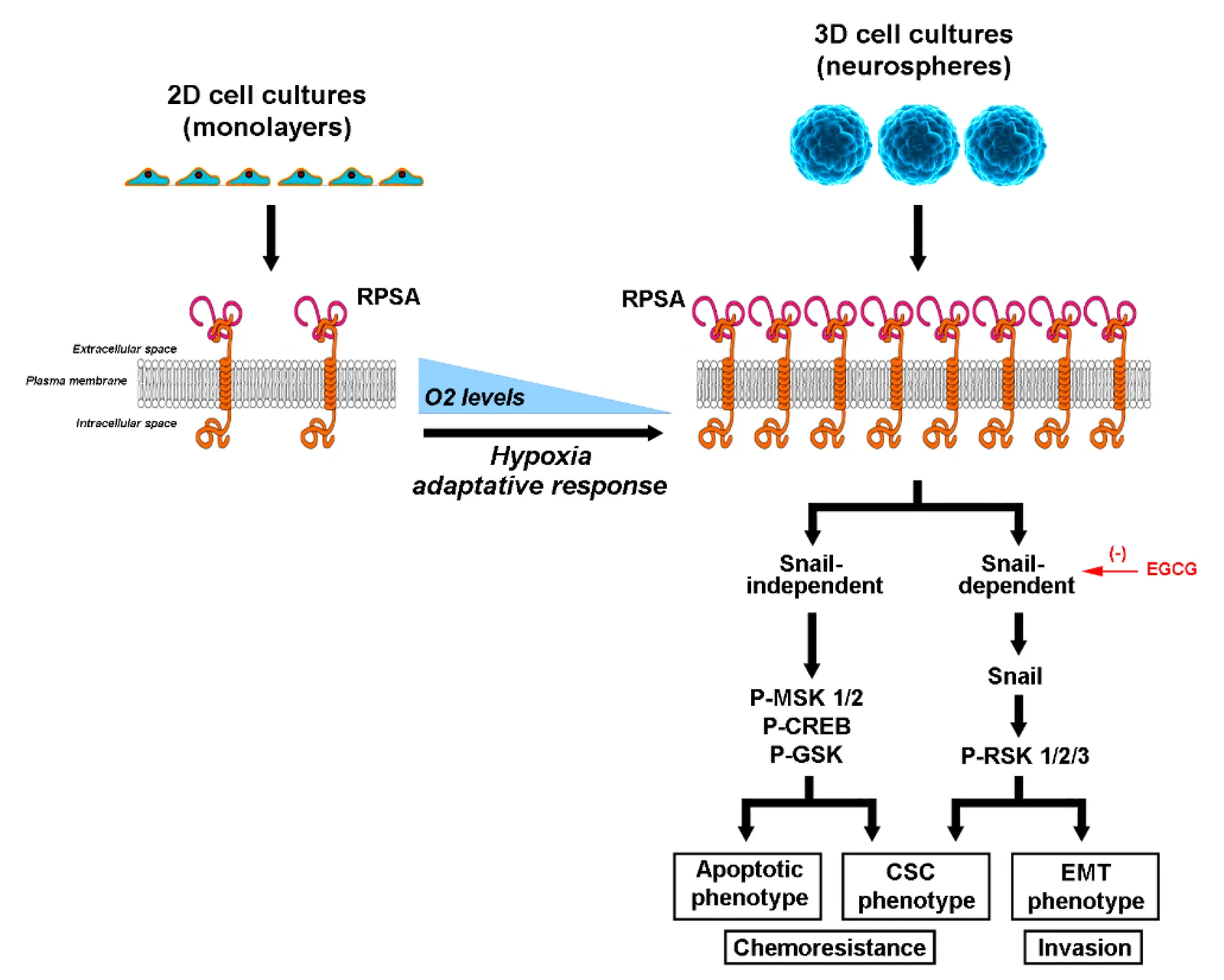
Schematic representation summarizing the crosstalk linking SNAIL to RPSA and the action of EGCG on the neurosphere molecular signature. U87 glioblastoma monolayers (2D cell cultures) can be cultured into 3D neurospheres to recapitulate the CSC invasive and chemo-resistant phenotypes through, in part, the induction of epithelial-to-mesenchymal (EMT) process and adaptative response to low O_2_ levels (hypoxia). Neurospheres express increased RPSA levels, contributing to both SNAIL-dependent and SNAIL-independent events. EGCG (red arrow) was demonstrated to target SNAIL-dependent signaling by reducing EMT biomarkers, and RSK phosphorylation, to impede spheroids growth and CSC phenotype. A potential adaptative apoptotic phenotype also involves SNAIL-independent signaling where RPSA regulates the phosphorylation status of MSK/CREB/GSK3α/β during spheroids formation. Altogether, current EGCG pharmacological intervention against acquiring of a CSC phenotype supports its chemopreventive properties.

Natural plants compounds have been reported as modulators of transition processes such as EMT [40], or such as those documented in adipose-derived mesenchymal stem/stromal cells where EGCG prevented the onset of an inflammatory and cancer-associated adipocyte-like phenotype in response to the triple-negative breast cancer secretome [41]. Regarding EGCG chemopreventive effects, here we demonstrate that the acquisition of a CSC molecular signature can be reversed consequent to the inhibition of a signaling axis linking RPSA to both SNAIL-dependent and -independent events, which in addition, leads to reduced spheroids size and stemness phenotype. As such, expression of both EMT biomarkers RPSA and SNAIL was induced in 3D neurospheres compared to 2D U87 glioblastoma cells monolayers as summarized herein (Figure 6). SNAIL involvement in EMT has been well documented, and cancer therapeutic opportunities envisioned [42]. EGCG intervention against SNAIL-mediated EMT events was also recently addressed in U87 glioblastoma cells [43] and ES2 ovarian cancer cells [44].

EMT is a trans-differentiation program and a key process in tumor progression that positively links the increased expansion of CSC and cells with stem-like properties [45]. Recent studies have provided insights into the molecular mechanisms underlying sustained EMT in CSC [46]. Interestingly, SNAIL contributes to several signaling pathways in EMT regulation, including those that involve STAT3 [47] and the Hippo pathway [48]. The regulation of EMT by SNAIL is also centered by several other signaling pathways, including primary mediators TGFβ, Notch, Wnt, TNFα, Hedgehog, and RTKs [49]. Here, we establish, for the first time to our knowledge, a signaling axis requiring the functions of RPSA upstream of SNAIL. Pertinent to our study, Notch3 was found to transactivate GSK3β and to inhibit EMT in a breast cancer cell model [50]. GSK3β regulates EMT and CSC properties in triple-negative breast cancer [51]. Although speculative, one can not preclude that, in our current model, a crosstalk involving MSK/CREB/GSK3α/β may rather regulate SNAIL upstream. As such, SNAIL is phosphorylated at Ser104/Ser107 and Ser96/Ser100 by casein kinase 1 and GSK3β, respectively [52,53]. Here, we also found that RSK phosphorylation appears to be a consequence of an upstream RPSA/SNAIL-dependent signaling which inhibition was shown to occur upon either RSPSA or SNAIL silencing, but also upon EGCG treatment. In accordance, preventing tumor recurrence through RSK inhibition resulted in the elimination of the CSC population in a TNBC cell model [54].

Finally, we highlight the increased levels in RPSA, also termed the 67-kDa laminin receptor (67LR) and correlate its expression to the acquired CSC phenotype in neurospheres. Interestingly, specific targeting of multiple myeloma cells has been linked to a high expression of the 67LR [55,56], which has been shown to be a cell surface receptor for EGCG binding [57]. The 67LR is also known to be overexpressed on the cell surface of various tumour cells, and expression level is strongly correlated with the risk of tumor invasion and metastasis [58], potentially regulating EMT [59].

Long-term expansion of neurospheres from GBM has not always been possible and continues to be a constraint, despite the neurosphere culture technique’s efficacy in enriching tumor-initiating cells from brain tumours. In fact, the success rate for glioblastoma stem cell (GSC) line isolation from any given tumour is surprisingly low (1–30%). In the meantime, some authors asserted that neurosphere assays for establishing GSC cell lines were equally effective, but they did not provide quantitative evaluation of the in vitro stemness phenotypes or a comparison of the in vivo tumorigenicity of cells from adherent and neurosphere tumour cultures. Some of the restrictions (like the rarity of true stem cells capable of prolonged self-renewal) need to be verified with regard to GSCs. Thus, even though neurosphere assays are a reliable and popular in vitro approach for obtaining and growing GSC, the aforementioned drawbacks will necessitate a rigorous assessment of their application in certain experimental contexts. Further research is required to better define the characteristics of the neoplastic spheres phenotype studied here because GSC neurospheres exhibit phenotypic characteristics different from those of their normal counterparts, such as a higher rate of proliferation and aberrant differentiation that results in cells co-stained for astrocytic and neuronal markers.

## 5. Conclusions

In conclusion, while 3D neurospheres assays are commonly used to uncover more relevant brain tumor biology than classical 2D culture conditions, one must acknowledge that such assays at this stage may still preclude direct clinical application. Here, we unravel a new RPSA/SNAIL original signaling axis regulating the global adaptative apoptotic, EMT, and CSC molecular signatures triggered upon 3D spheroids cultures, which can be prevented through the pharmacological actions of a diet-derived polyphenol such as EGCG.

## Data Availability

The raw data supporting the conclusions of this article are available from the authors upon reasonable request.

## Supplementary Materials

The following supporting information can be downloaded at: https://www.mdpi.com/article/10.3390/cancers14235944/s1, Figure S1: EGCG lack of cytotoxicity; Figure S2: Uncropped WB of Figure 1; Figure S3: Uncropped WB of Figure 3; Figure S4: WB of Figure 4; Figure S5: Uncropped WB of Figure 4: Figure S6: Uncropped WB of Figure 5.

## Abbreviations

CSC, Cancer stem cells; CREB, cAMP response element-binding protein; EGCG, Epigallocatechion-3-gallate; EMT, epithelial-to-mesenchymal transition; FN, Fibronectin; GBM, Glioblastoma; GSC, Glioblastoma stem cells; GSEA, gene set enrichment analysis; GSK3, Glycogen synthase kinase-3; LGG, low grade glioma; RPSA, 37/67 kDa laminin-1 receptor ribosomal protein SA; RSK, 90 kDa ribosomal S6 kinase.
