# Correction: Gresseau et al. A Signaling Crosstalk Links SNAIL to the 37/67 kDa Laminin-1 Receptor Ribosomal Protein SA and Regulates the Acquisition of a Cancer Stem Cell Molecular Signature in U87 Glioblastoma Neurospheres. *Cancers* 2022, *14*, 5944

**DOI:** 10.3390/cancers16051065

**Published:** 2024-03-06

**Authors:** Loraine Gresseau, Marie-Eve Roy, Stéphanie Duhamel, Borhane Annabi

**Affiliations:** 1 Laboratoire d’Oncologie Moléculaire, Département de Chimie, and CERMO-FC, Université du Québec à Montréal, Montreal, QC H3C 3J7, Canada; 2 Goodman Cancer Institute, McGill University, Montreal, QC H3A 0G4, Canada

## Error in Figure

In the original publication [1], there was an honest mistake in ***Figure 4D (P-AKT panel)*** as published. **The P-AKT expression was erroneously duplicated from Figure 5D.** The corrected Figure 4D appears below. The authors apologize for any inconvenience caused and state that the scientific conclusions are unaffected. This correction was approved by the Academic Editor. The original publication has also been updated.

**Figure 4:**
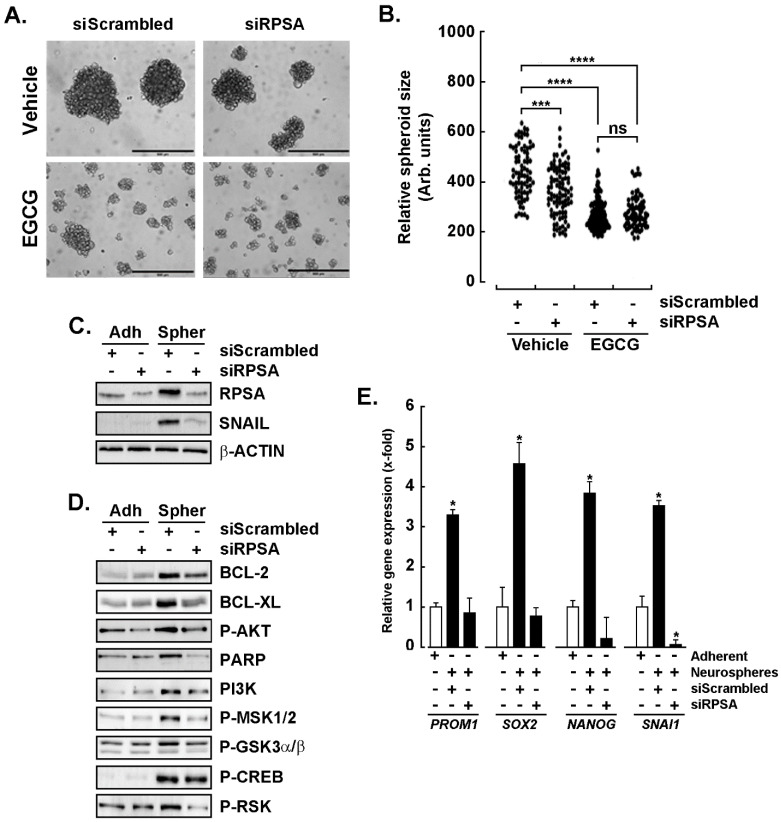
Repression of RPSA alters spheroids formation and prevents the acquisition of a cancer stem cell phenotype. (**A**) U87 glioblastoma monolayers were transiently transfected with a scrambled sequence (siScrambled) or with a siRNA directed against RPSA (siRPSA). Next, the cells were cultured with the Tumorsphere Medium Xf with Supplement Mix for 72 h in the absence or presence of 30 μM EGCG. Representative phase contrast pictures of the spheroids formed were taken, and (**B**) spheroid size was quantified as described in the Methods section. (*** = 0.001 ≥ *p*; **** = 0.0001 ≥ *p*; ns = non significant). Cell lysates were harvested from adherent, or spheroids obtained at 72 h and upon transient siScrambled or siRPSA transfection. Western blot analysis of (**C**) EMT markers RPSA, SNAIL, and β-Actin, as well as the indicated (**D**) apoptosis and transducing intermediates expression was performed as described in the Methods section. Representative blots for each marker are shown from three independent experiments. (**E**) Total RNA was extracted from adherent or neurospheres and RT-qPCR analysis was used to assess the expression of CSC markers (*PROM1*, *SOX2*, and *NANOG*), and EMT marker *SNAI1*. Probability values of 0.05 were judged significant and indicated as (*).
