# Correction: Gresseau et al. A Signaling Crosstalk Links SNAIL to the 37/67 kDa Laminin-1 Receptor Ribosomal Protein SA and Regulates the Acquisition of a Cancer Stem Cell Molecular Signature in U87 Glioblastoma Neurospheres. *Cancers* 2022, *14*, 5944

**DOI:** 10.3390/cancers18152491

**Published:** 2026-08-04

**Authors:** Loraine Gresseau, Marie-Eve Roy, Stéphanie Duhamel, Borhane Annabi

**Affiliations:** 1 Laboratoire d’Oncologie Moléculaire, Département de Chimie, and CERMO-FC, Université du Québec à Montréal, Montreal, QC H3C 3J7, Canada; 2 Goodman Cancer Institute, McGill University, Montreal, QC H3A 0G4, Canada

## Error in Figure 3

In the original publication [1], there was a mistake in Figure 3B as published. Fibronectin and RPSA labeling must be interchanged in order to be aligned with the raw data made available in the ‘Uncropped WB Figure S3’ blots provided in the supplemental data. The corrected Figure 3B appears below. The authors state that the scientific conclusions are unaffected. This correction was approved by the Academic Editor. The original publication has also been updated.

## Error in the Data Availability Statement

There were mistakes in the Data Availability Statement as published: All data generated or analyzed during this study are included in this published article.

The corrected statement appears now as follows: The raw data supporting the conclusions of this article are available from the authors upon reasonable request.

The authors state that the scientific conclusions are unaffected. This correction was approved by the Academic Editor. The original publication has also been updated.

## Figures and Tables

**Figure 3:**
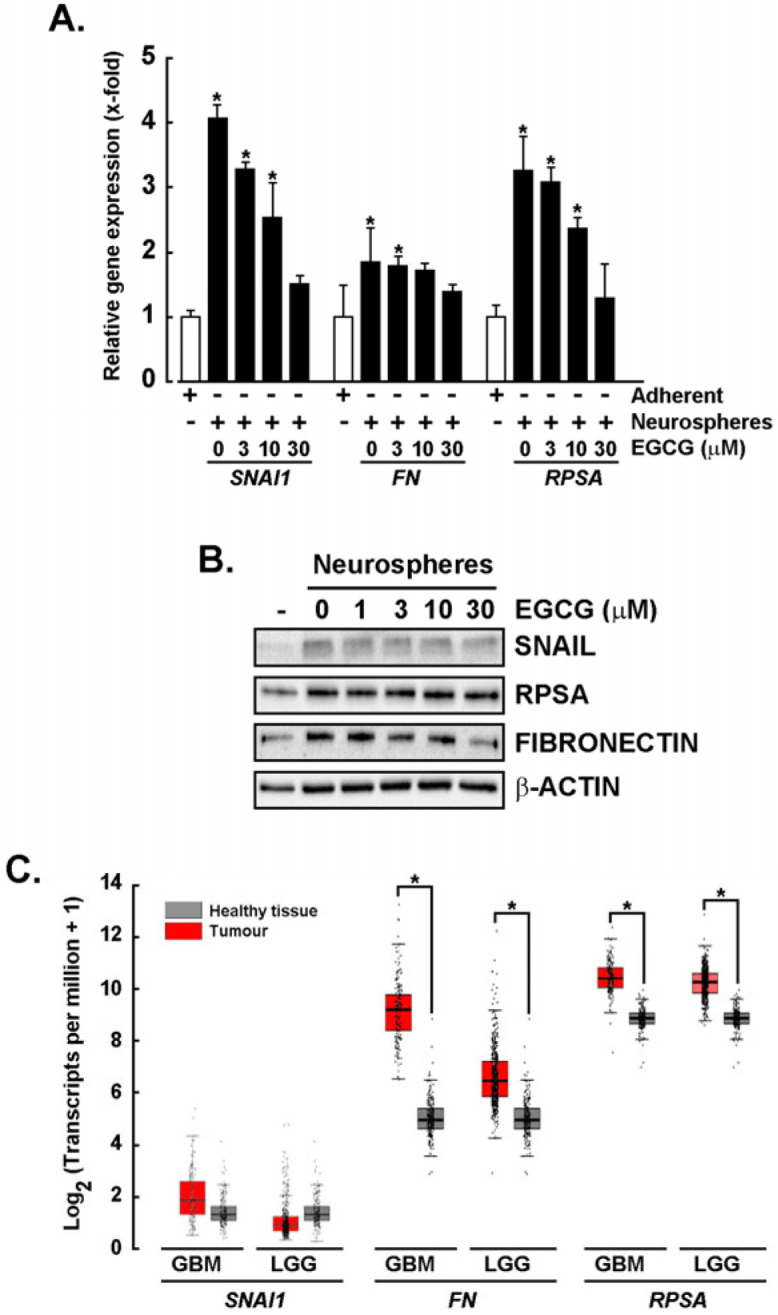
EGCG alters neurospheres’ epithelial-to-mesenchymal transition phenotype. (**A**) U87 glioblastoma monolayers were cultured with the Tumorsphere Medium Xf with SupplementMix to generate spheroids for 72 h in the absence or presence of increasing EGCG concentrations. Total RNA was extracted from adherent or neurospheres, and RT-qPCR analysis was used to assess the expression of EMT markers SNAIL, Fibronectin (FN), and RPSA (67 kDa Laminin Receptor). (**B**) Cell lysates were harvested from adherent (Adh), or neurospheres obtained at 72 h. Western blot analysis of protein expression was performed as described in the Methods section of the described markers. Representative blots for each marker are shown from three independent experiments. (**C**) In silico analysis of *SNAI1*, *FN*, and *RPSA* transcript levels was performed on clinical samples from GBM, and low-grade glioma (LGG) and compared to healthy tissue as described in the Methods section. Probability values of 0.05 were judged significant and indicated in the figure as (*).
