# Supplementary material for: A Signaling Crosstalk Links SNAIL to the 37/67 kDa Laminin-1 Receptor Ribosomal Protein SA and Regulates the Acquisition of a Cancer Stem Cell Molecular Signature in U87 Glioblastoma Neurospheres
Source: Cancers (Basel). 2022 Nov 30;14(23):5944. doi: 10.3390/cancers14235944 (PMC9738384; doi:10.3390/cancers14235944)
Supplement: Supplementary file 1 [file cancers-14-05944-s001.zip › Supplemental.pptx]

## Slide 1
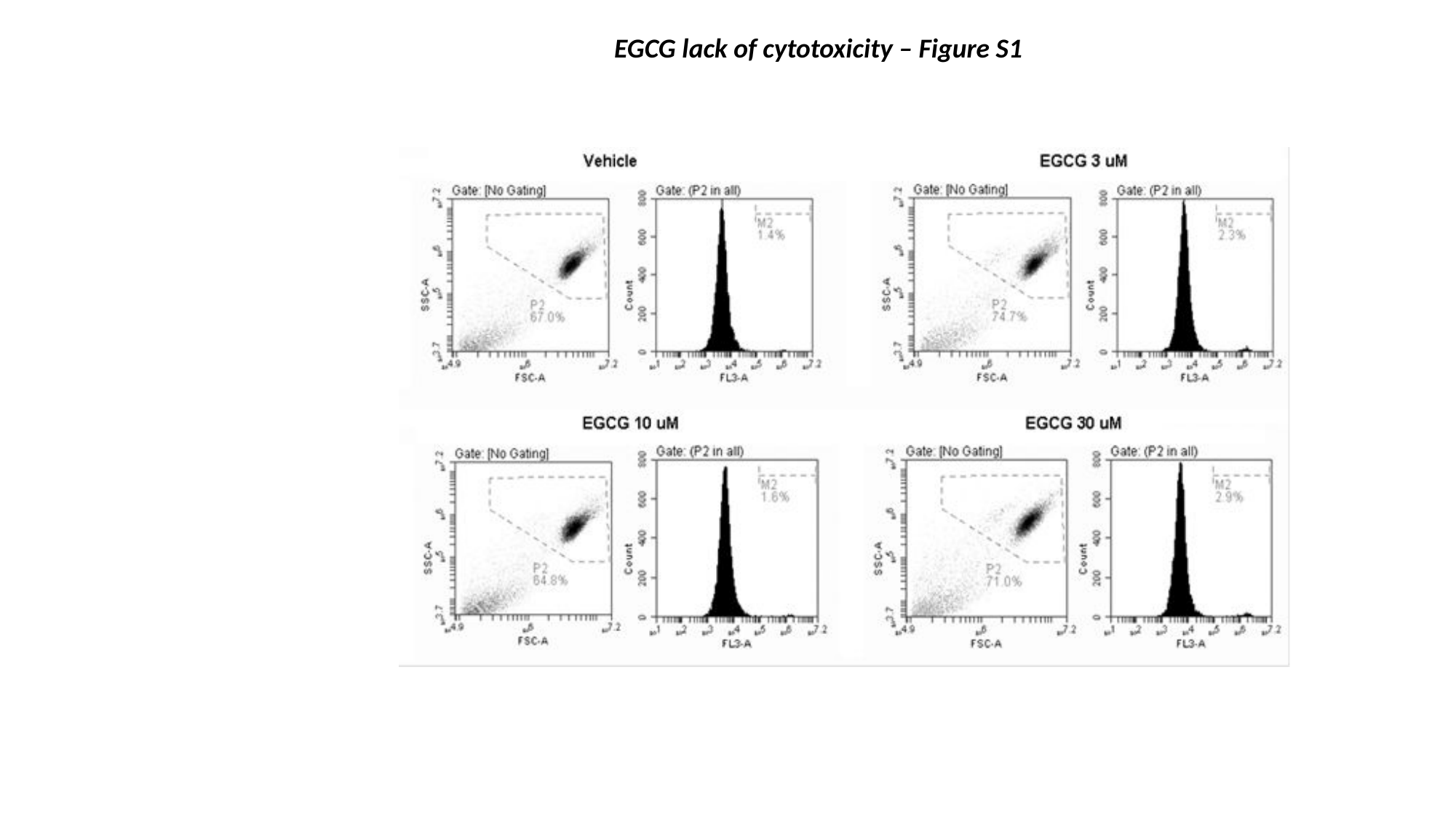

EGCG lack of cytotoxicity – Figure S1

## Slide 2
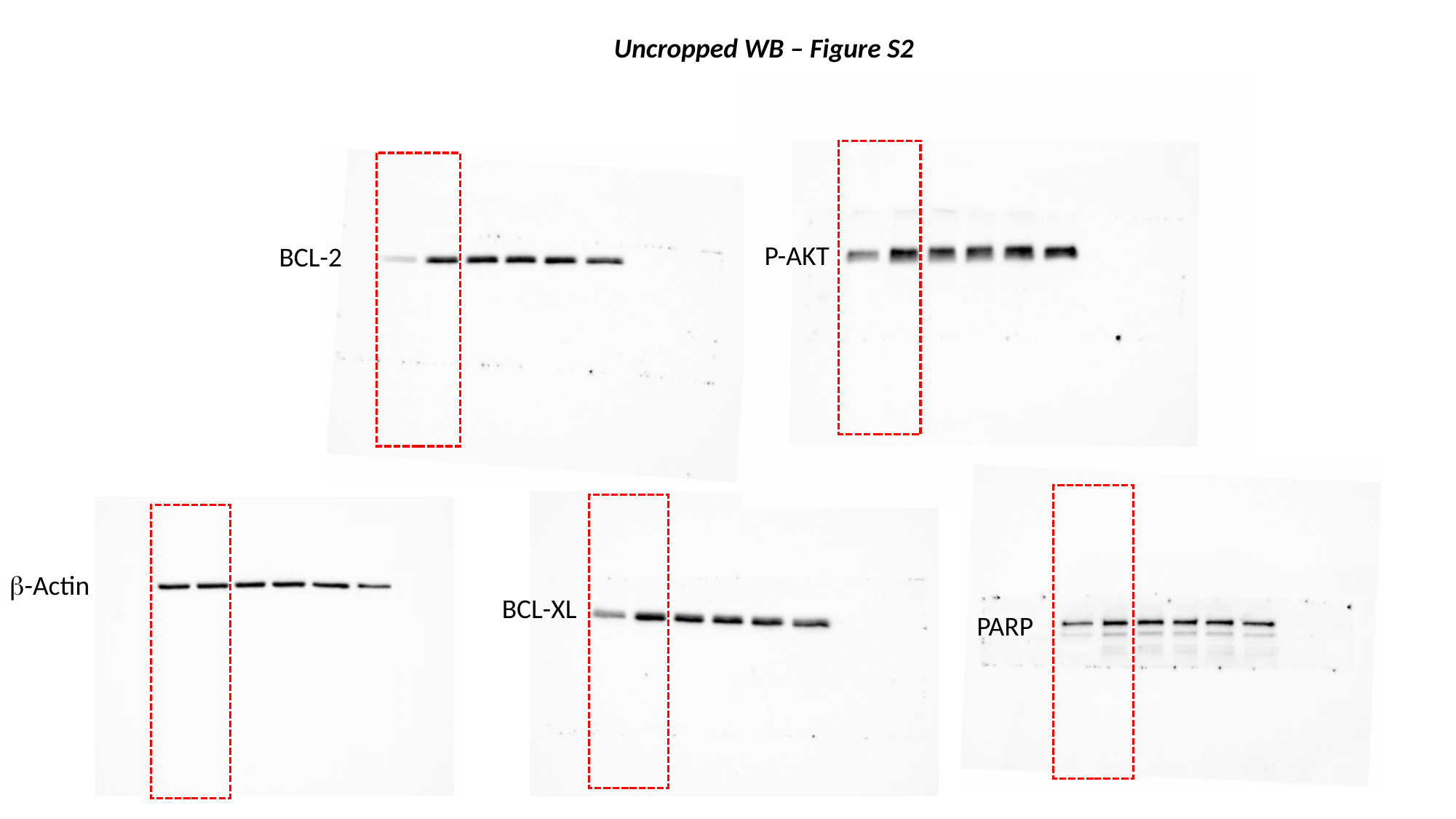

Uncropped WB – Figure S2
P-AKT
BCL-2
b-Actin
BCL-XL
PARP

## Slide 3
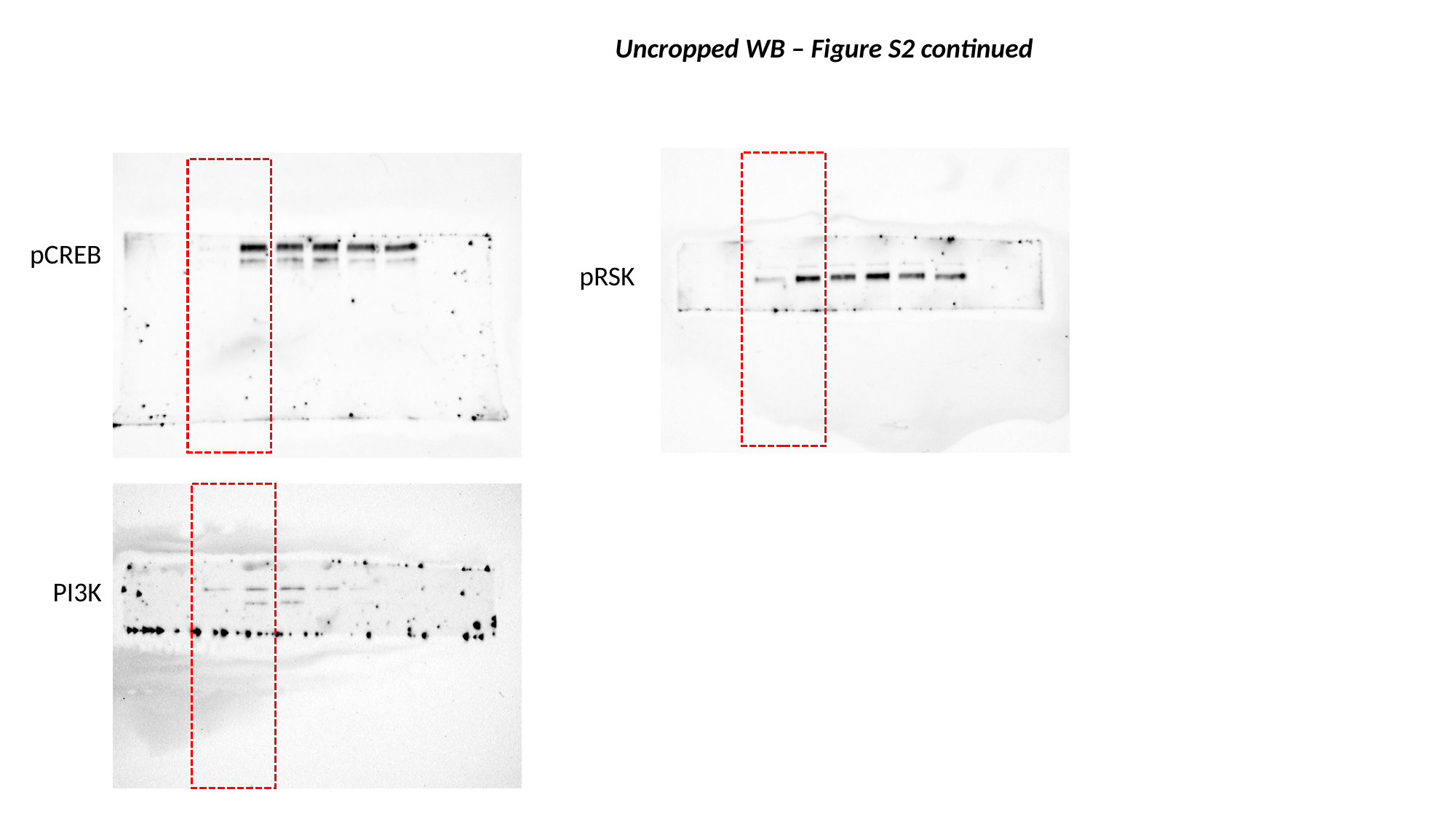

Uncropped WB – Figure S2 continued
pCREB
pRSK
PI3K

## Slide 4
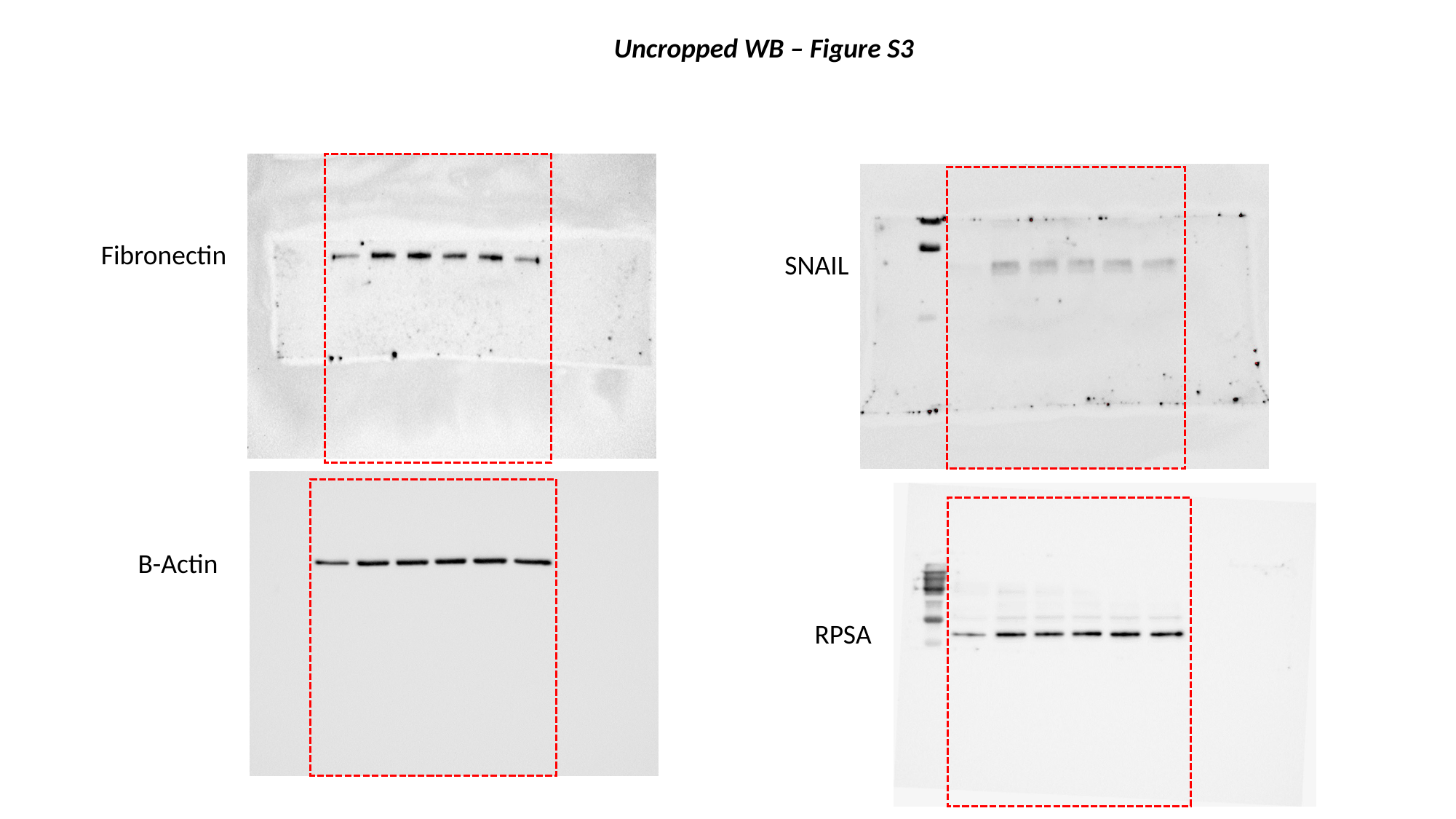

Uncropped WB – Figure S3
Fibronectin
SNAIL
B-Actin
RPSA

## Slide 5
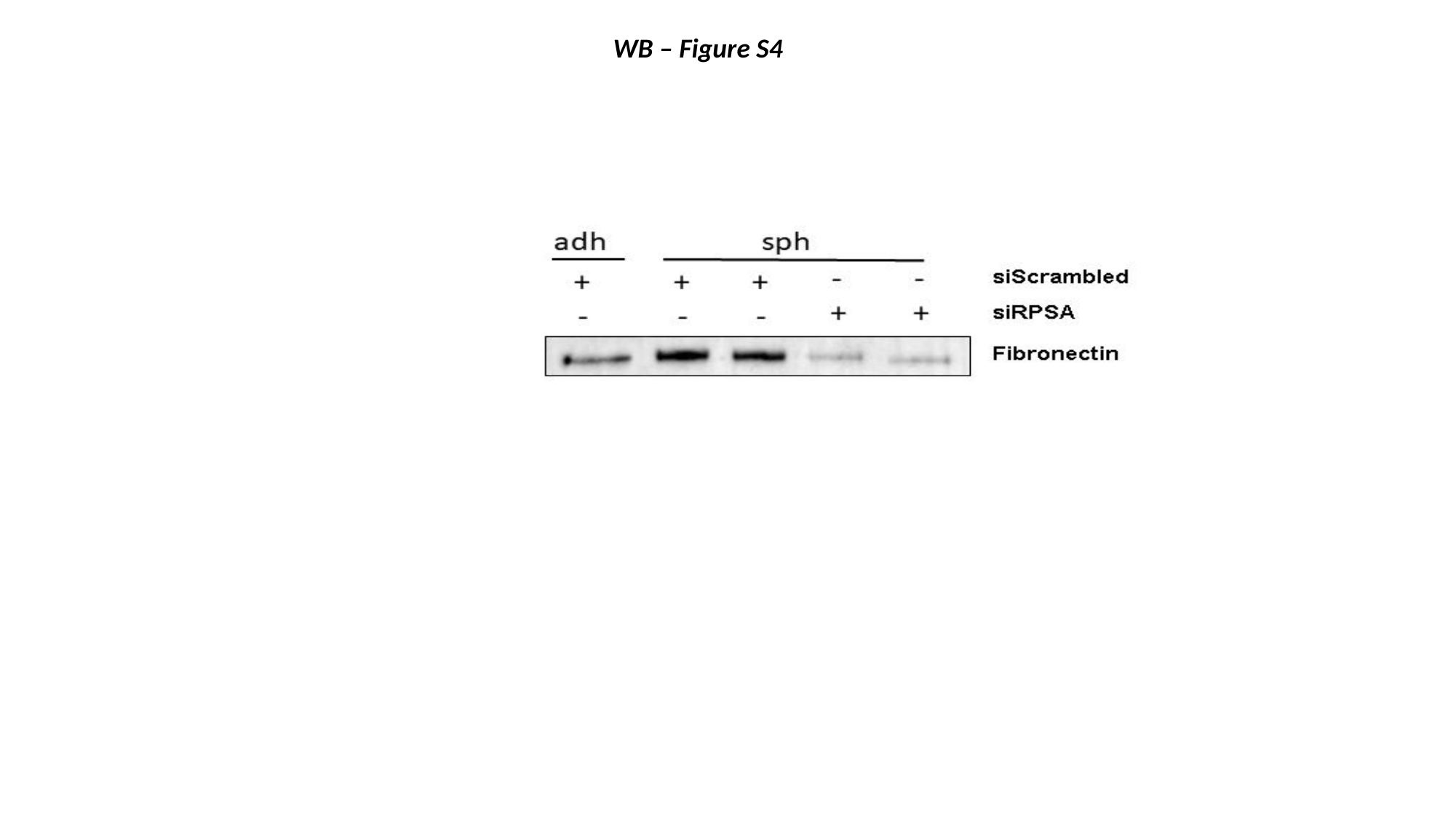

WB – Figure S4

## Slide 6
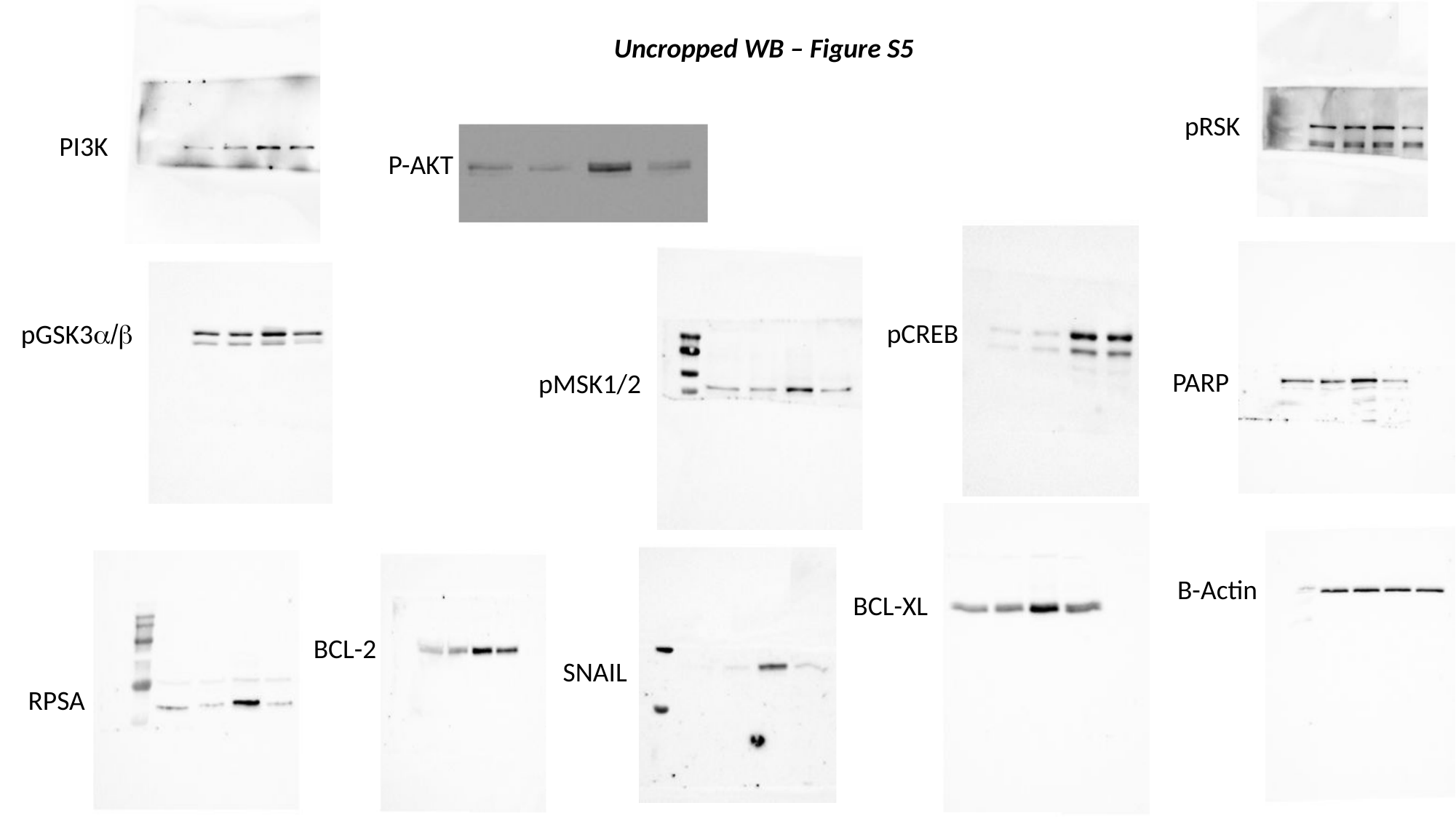

Uncropped WB – Figure S5
pRSK
PI3K
P-AKT
pCREB
pGSK3a/b
PARP
pMSK1/2
B-Actin
BCL-XL
BCL-2
SNAIL
RPSA

## Slide 7
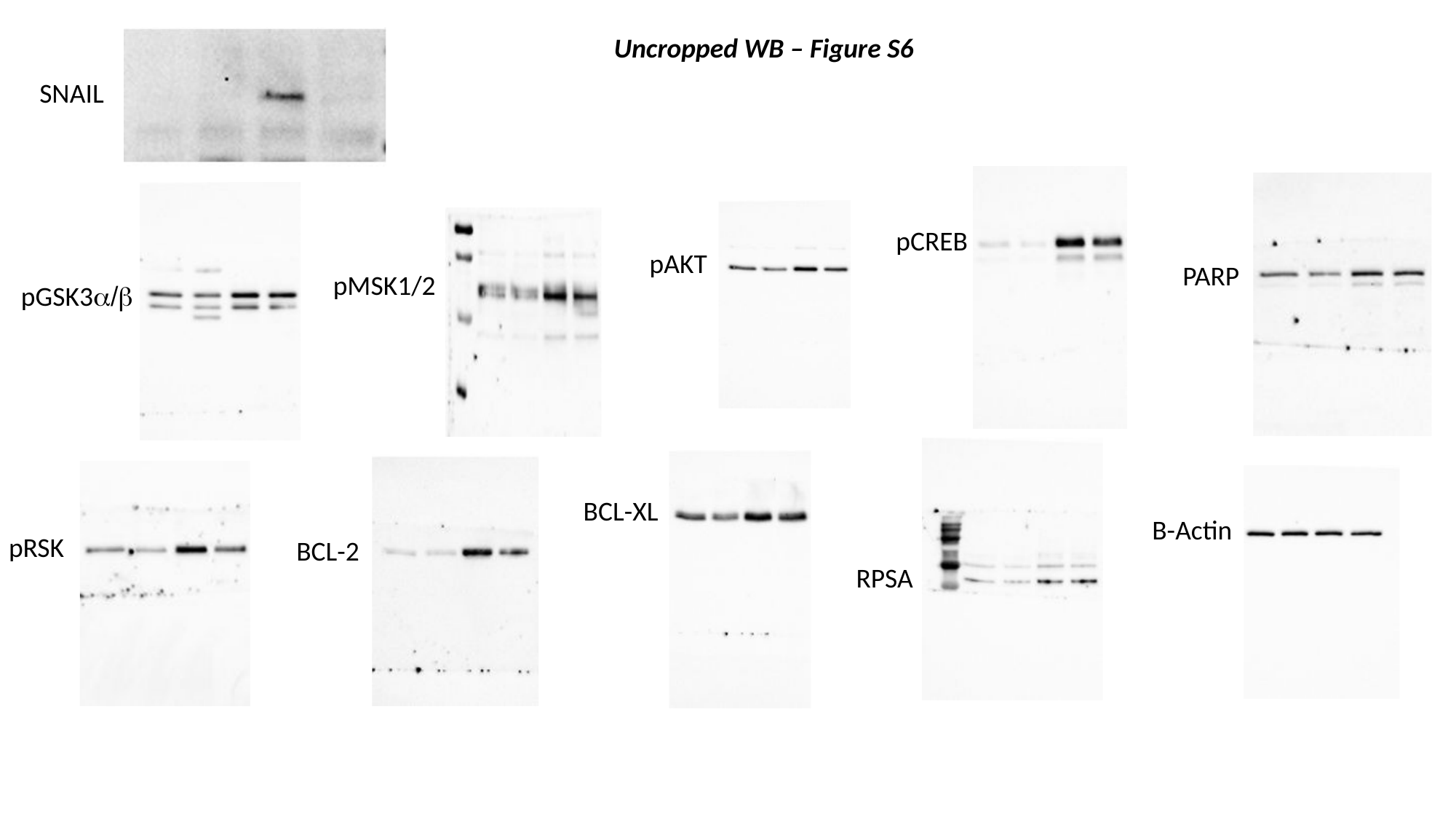

Uncropped WB – Figure S6
SNAIL
pCREB
pAKT
PARP
pMSK1/2
pGSK3a/b
BCL-XL
B-Actin
pRSK
BCL-2
RPSA
